# Assessment of pathologic increase in liver stiffness enables earlier diagnosis of CFLD: Results from a prospective longitudinal cohort study

**DOI:** 10.1371/journal.pone.0178784

**Published:** 2017-06-02

**Authors:** Victoria Klotter, Caroline Gunchick, Enno Siemers, Timo Rath, Helge Hudel, Lutz Naehrlich, Martin Roderfeld, Elke Roeb

**Affiliations:** 1 Department of Internal Medicine, Division of Gastroenterology, Justus-Liebig-University, Giessen, Germany; 2 Department of Medicine 1, University of Erlangen-Nuremberg, Erlangen, Germany; 3 Institute for Medical Informatics, Justus-Liebig-University, Giessen, Germany; 4 Department of Pediatrics, Division of Pulmonology, Justus-Liebig-University, Giessen, Germany; Medizinische Fakultat der RWTH Aachen, GERMANY

## Abstract

About 30% of patients with Cystic Fibrosis (CF) develop CF-associated liver disease (CFLD). Recent studies have shown that transient elastography (TE), as a method to quantify liver stiffness, allows non-invasive diagnosis of CFLD in adults and children with CF. Within this study we aimed to prospectively identify patients at risk for development of CFLD by longitudinal analysis of liver stiffness and fibrosis scores in a 5-year follow-up. 36 pediatric and 16 adult patients with initial liver stiffness below the cut-off value indicative of CFLD (6.3 kPa) were examined by transient elastography for 4–5 years. TE, APRI-, and FIB-4-scores were assessed and compared by Kruskal-Wallis test and receiver operating characteristic (ROC)-analysis. Frequencies were compared by Chi^2^-test. Among the 36 patients participating in this study, a subgroup of 9 patients developed liver stiffness >6.3 kPa after 4–5 years with an increase of ΔTE >0.38 kPa/a (the group with increasing liver stiffness was labelled TE_inc_). APRI- and FIB-4 scores confirmed the rationale for grouping. The frequency of CFLD assessed by conventional diagnosis was significantly higher in TE_inc_-group compared to the control group (TE_norm_). None of the adult CF patients matched criteria for TE_inc_-group. For the first time it was shown that the non-invasive longitudinal assessment of TE allows identification of patients with progression of CFLD in a subgroup of juvenile but not in adult CF patients. Comparing TE to conventional fibrosis-scores underlined the strength of the continuous assessment of liver stiffness for the exact diagnosis of progressive CFLD. The newly described cut-off for pathologic increase of liver stiffness, ΔTE_cutoff_ = 0.38kPa/a, might enable to detect developing CFLD using consequent follow up TE measurements before reaching the level of stiffness indicating established CFLD. Nevertheless, the limited size of the analyzed cohort should encourage a prospective, multi-center, long term follow up study to confirm the suggested cut-off for the rise in liver stiffness.

## Introduction

Cystic fibrosis (CF) is the most common multisystem autosomal recessive genetic disorder with a high morbidity and mortality in Caucasian population with an incidence of approximately 1 of 3500 births worldwide [1]. Despite continuous improvements in the treatment of CF patients, the disease is associated with a high annual mortality rate of 1.6% with a median age at death of 29.1 years [1]. According to the current Cystic Fibrosis Foundation Patient Registry, Cystic Fibrosis associated liver disease (CFLD) causes 2.5% of the overall mortality of CF patients and represents the third most common cause of death in CF patients [1]. To date, liver biopsy remains the gold standard for the assessment of the severity of liver diseases such as fibrosis and cirrhosis [2]. However, it is an invasive and painful procedure with rare but potentially life threatening complications which is a limitation to its acceptance and repetition, especially in children. Hence, a wide range of research has been focused on the development of non-invasive methods to diagnose the CFLD. Several studies were able to prove that quantification of liver stiffness by transient elastography (TE) is a novel, non-invasive method for the diagnosis of CFLD [3–6]. However, all studies evaluating TE for the non-invasive diagnosis of CFLD are retrospective and cross sectional. A prospective longitudinal non-invasive CFLD evaluation has not been reported so far. Longitudinal data of liver stiffness from patients with developing CFLD might allow to specify a cut-off for the rise in liver stiffness. Consequently, this cut-off might be a valuable diagnostic tool to identify developing CFLD before reaching the level of stiffness which is known for established CFLD. The aim of this study was to define a cut-off for the rise in liver stiffness in a prospective longitudinal cohort study. Within this study, we prospectively assessed in a 5-year follow-up whether transient elastography allows the identification of patients at risk for the progression of liver disease. For this purpose, patients received an annual quantification of liver stiffness by TE and the results were compared with two well-known fibrosis scores, APRI and FIB-4. Our results show that this approach has the potential to identify CF patients at risk of developing progression of CF liver disease.

## Materials and methods

### Study subjects and design

This prospective 5-years follow-up study was performed during the period of 2009–2015. It has been conducted according to the principles of the Declaration of Helsinki and approved by the ethics committee of the medical faculty of the Justus-Liebig-University Giessen (35392 Giessen, Germany) with the approval No. 75/09.

All participating adult patients and the parents of the pediatric patients received, signed and agreed with the written informed consent for the procedure for the upcoming period of minimum five years in all cases. The treatment meets all requirements of the European and U.S. guidelines [7].

The diagnosis of CF was an essential prerequisite for participating in this study and was established by the sweat chloride test with application of pilocarpine and the positive gene mutation tests with elevated immunoreactive trypsinogen (IRT) in all subjects.

In total, 173 out-patient positive cystic fibrosis (CF) patients, 85 males and 88 females, with a median age of 13 (2–47), were treated at the Department of Internal Medicine at the University Hospital Giessen. The cohort was classified into patients with liver disease (CFLD) on the one hand or without liver disease (CFnoLD) on the other hand according to the clinical guideline criteria published by Debray in 2011 [8]. They are defined as presenting at least two of the following conditions for CFLD on at least two consecutive examinations spanning a one-year period:

- hepatomegaly with or without a splenomegaly
- two abnormal serum liver enzyme levels (ALT, AST, γ-GT > upper limit of normal)
- other ultrasound parameters of liver abnormalities than hepatomegaly such as varied echogenicity, increased density, nodules, rough liver margins, evidence for portal hypertension (i.e. dilatation, splenomegaly, ascites…)

Patients with other diagnosed causes for chronic hepatobiliary diseases were excluded of this study [8].

All patients underwent clinical examination at intervals of approximately once a year.

In addition, each patient received regular haematological and biochemical investigation as well as a pulmonary function test (FEV1, VC) [9,10].

Results were computed to **APRI** (Aspartate Aminotransferase to Platelet Ratio = AST/upper limit of normal AST x 100/Platelet Count (10^9^/l) and **FIB-4** (Fibrosis-4 = Age (years) x AST (U/l) / Platelets 10^9^/L x 3 ALT (U/l)) which are validated to detect fibrosis and predict cystic fibrosis liver disease (CFLD), as described by Leung et al. [2].

Furthermore, all patients underwent a regular abdominal ultrasound examination (Philips HD 11X E) as well as an examination with the transient elastography (TE) with FibroScan®.

Our study focuses on juvenile patients, whose valid and complete measures were conducted over 4–5 years. Those with an already existing high liver stiffness (above the cut-off) were excluded from this study.

### Transient elastography

Assessment of liver stiffness was performed as described before [6].

### Statistical analysis

After collecting the patients´ demographic and clinical characteristics of up to 5 years, IBM SPSS Statistics 22.0 (SPSS Inc., Chicago, III) was used to summarize and perform the statistical analysis.

Differences in the TE values, ΔTE, median TE (0–5), as well as APRI- and FIB-4-score were assessed and compared in multiple tests using the Kruskal-Wallis-test.

The values of the liver stiffness measured with TE, APRI- and FIB-4-score are visualized in Box- and- Whisker-Plots. Outliers (o) define the values deviating from the box at 1.5- to 3-fold interquartile range. Significant and highly significant differences are pointed out (*p≤0.05, **p≤0.001). Pearson’s Chi-square test compares and differentiates between the frequency of CFnoLD and CFLD in the two subgroups.

The diagnostic performance and accuracy of TE, APRI, FIB-4 and ALT were assessed by using the receiver operating characteristics (ROC)-analysis.

## Results

**Fig 1:**
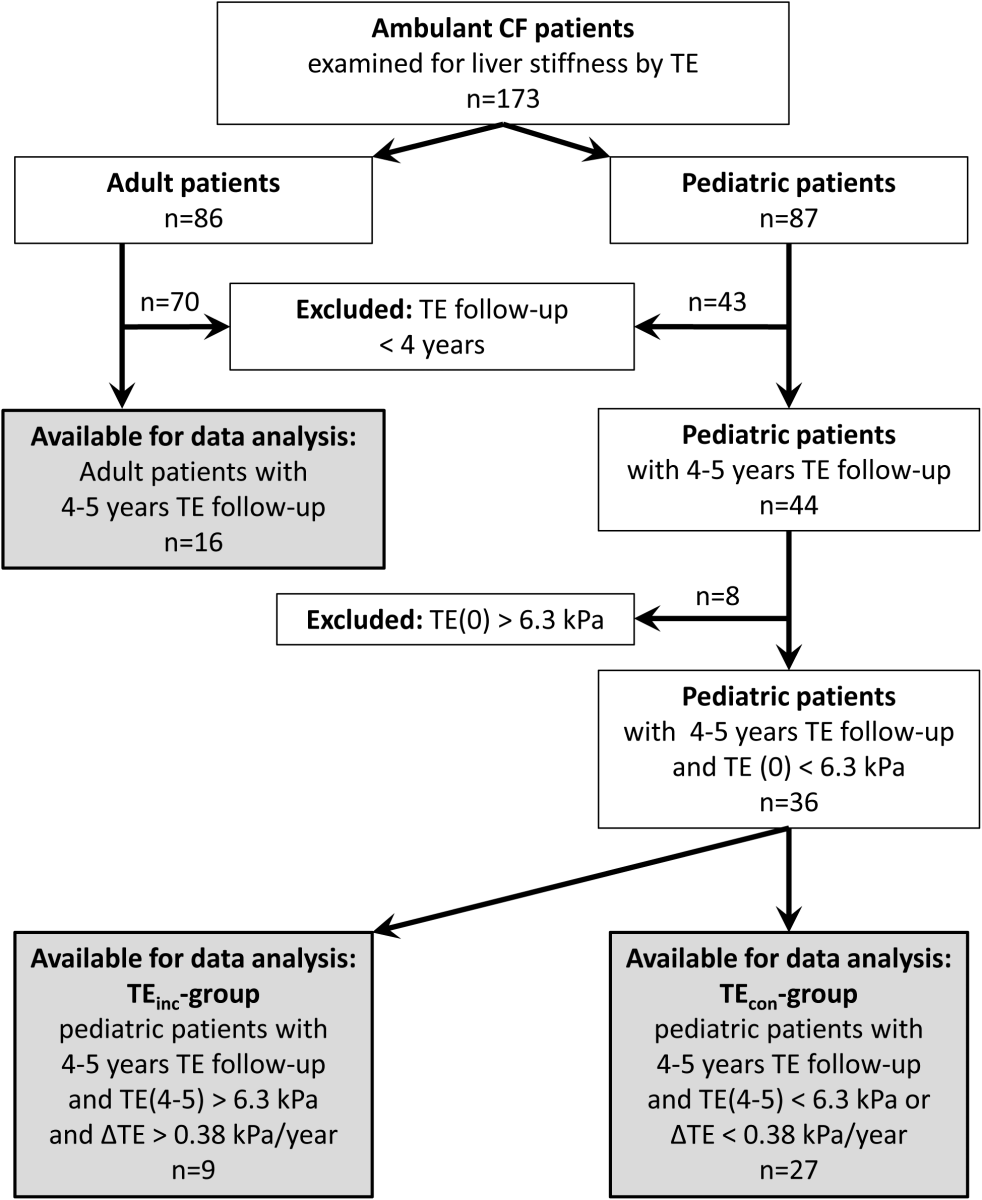
Derivation of study cohort. TE = Transient elastography.

For 60 of the 173 CF patients, including 16 adults and 44 children, follow-up data with initial and regularly consecutive TE over 4–5 years and quantification of APRI- and FIB-4 scores were available (Fig 1). 21 of the 44 pediatric patients were diagnosed with CFLD as defined by clinical guidelines criteria [8] during the follow-up. Compared to children without CFLD, those with CFLD had significantly elevated mean values of liver stiffness, serum ALT, and APRI over the follow-up while FIB-4 was not altered: TE(noCFLD) = 4.5±0.16 kPa and TE(CFLD) = 8.2±1.6 kPa with *p*<0.001, ALT(CFnoLD) = 24.7±1.6 U/l and ALT(CFLD) = 39.2±4.7 U/l with *p*<0.003, APRI(CFnoLD) = 0.20±0.02 and APRI(CFLD) = 0.36±0.08 with *p* = 0.007. The values were calculated as means of all measurements at all time points during the follow-up. In theROC-analysis, the following diagnostic accuracies of TE, serum ALT, APRI- and FIB-4 scores for the distinction between CFLD and CFnoLD were calculated: AUC(TE) = 0.91±0.05 with *p*<0.001, AUC(ALT) = 0.76±0.08 with *p* = 0.003, AUC(APRI) = 0.74±0.08 with *p* = 0.007, AUC(FIB-4) = 0.62±0.09 with *p* = 0.17. These data are consistent with the results of previous studies [2,5,6]. Based on these results, we then aimed to analyze whether TE or non-invasive fibrosis scores might be able to identify CF patients at risk for developing CFLD during the course of the disease.

This prospective identification of pediatric patients at risk for the development of CFLD, analyzing the liver stiffness and the fibrosis scores longitudinally, required the following stratification (Table 1): 36 children of the 44 pediatric patients had an initial liver stiffness TE(0) below the threshold of 6.3 kPa which has been shown to be the optimum for the distinction of patients with and without CFLD [5].

**Table 1:** Demographic and clinical data of the pediatric cystic fibrosis patient cohort.

Demographic and clinical data	TE_con_		TE_inc_	
Male (n/%)	18/66.7%		8/88.9%	
Female (n/%)	9/33.3%		1/11.1%	
Examination	initial	last	initial	last
**Age (years)**	10.0 (11) ±4.8	14.7 (16) ±4.7	10 (10) ±3.9	14.9 (14) ±4.0
Range	2–17	7–22	4–17	9–22
**BMI (kg/m**^**2**^**)**	16.7 (16.5) ±2.7	19.0 (18.1) ±3.4	15.9 (16.3) ±1.4	18.3 (18.9) ±1.8
**Liver Stiffness (kPa)**	4.66 (4.5) ±0.85	4.5 (4.3) ±0.74	4.4 (4.3) ±0.55	8.64 (8.2) ±1.71
Range	3.3–6.3	3.2–5.8	3.8–5.4	6.8–12.2
**ALT (U/l)**	25.1 (21) ±15.7	34.3 (26) ±27.5	44.3 (22) ±58.4	59.8 (32) ±65.2
Range	10–88	11–144	11–208	22–240
**AST (U/l)**	26.0 (25) ±7.1	37.0 (25) ±55.4	38.3 (29) ±19.8	57.9 (33) ±68.2
Range	15–46	14–316	20–84	23–248
**APRI**	0.19 (0.18) ±0.09	0.3 (0.2) ±0.39	0.31 (0.22) ±0.19	0.46 (0.28) ±0.45
Range	0.09–0.52	0.1–2.23	0.12–0.73	0.18–1.71
**FIB-4**	0.16 (0.15) ±0.08	0.28 (0.25) ±0.26	0.2 (0.17) ±0.08	0.32 (0.3) ±0.11
Range	0.03–0.34	0.1–1.56	0.07–0.34	0.19–0.62
**Platelet count (G/l)**	352 (333) ±99	301 (294) ±53	333 (329) ±48	297 (300) ±53
Range	220–757	217–405	245–406	201–363
**AP(U/l)**	254 (248) ±64	209 (184) ±119	260 (251) ±56	264 (279) ±76
Range	122–394	0.1–517	162–346	141–344
**Bilirubin**	0.43 (0.3) ±0.27	0.42 (0.5) ±0.17	0.33 (0.3) ±0.12	0.43 (0.5) ±0.09
Range	0.1–1	0.1–0.6	0.2–0.6	0.3–0.5
**Albumin (g/dl)**	45.3 (45.5) ±1.8	45.0 (46) ±2.8	42.1 (41) ±2.4	44.1 (44.2) ±2.6
Range	42–48	38–50	39–47	39–48
**PTT (%)**	34.8 (33.5) ±4.3	34.8 (34) ±2.9	34 (34) ±2.7	33 (3) ±1.6
Range	29–44	31–39	29–39	31–35
**γ-GT (U/l)**	11.5 (12) ±4.8	15.1 (13) ±8.3	16.1 (19) ±13.4	48.6 (25) ±71.3
Range	5–26	5–40	8–53	8–248
**Ferritin (µg/l)**	24.7 (21.5) ±10.6	35.1 (32) ±18.7	29.4 (24) ±9.9	37.8 (33) ±21.8
Range	11–50	6–98	17–45	6–70

Mean (median) ± SD and range is depicted, Abbreviations: SD = standard deviation; BMI = body- mass- index; TE = transient elastography; TE_inc_ = group of children with increasing liver stiffness, TE_con_ = control group of children without increasing liver stiffness; ALT = alanin- aminotransferase; AST = aspartate- aminotransferase; APRI = Aspartate Aminotransferase to Platelet Ratio Index; FIB-4 = Fibrosis-4 index; AP = alkaline phosphatase; PTT = partial thromboplastin time; γ-GT = γ–glutamyltransferase

**Fig 2:**
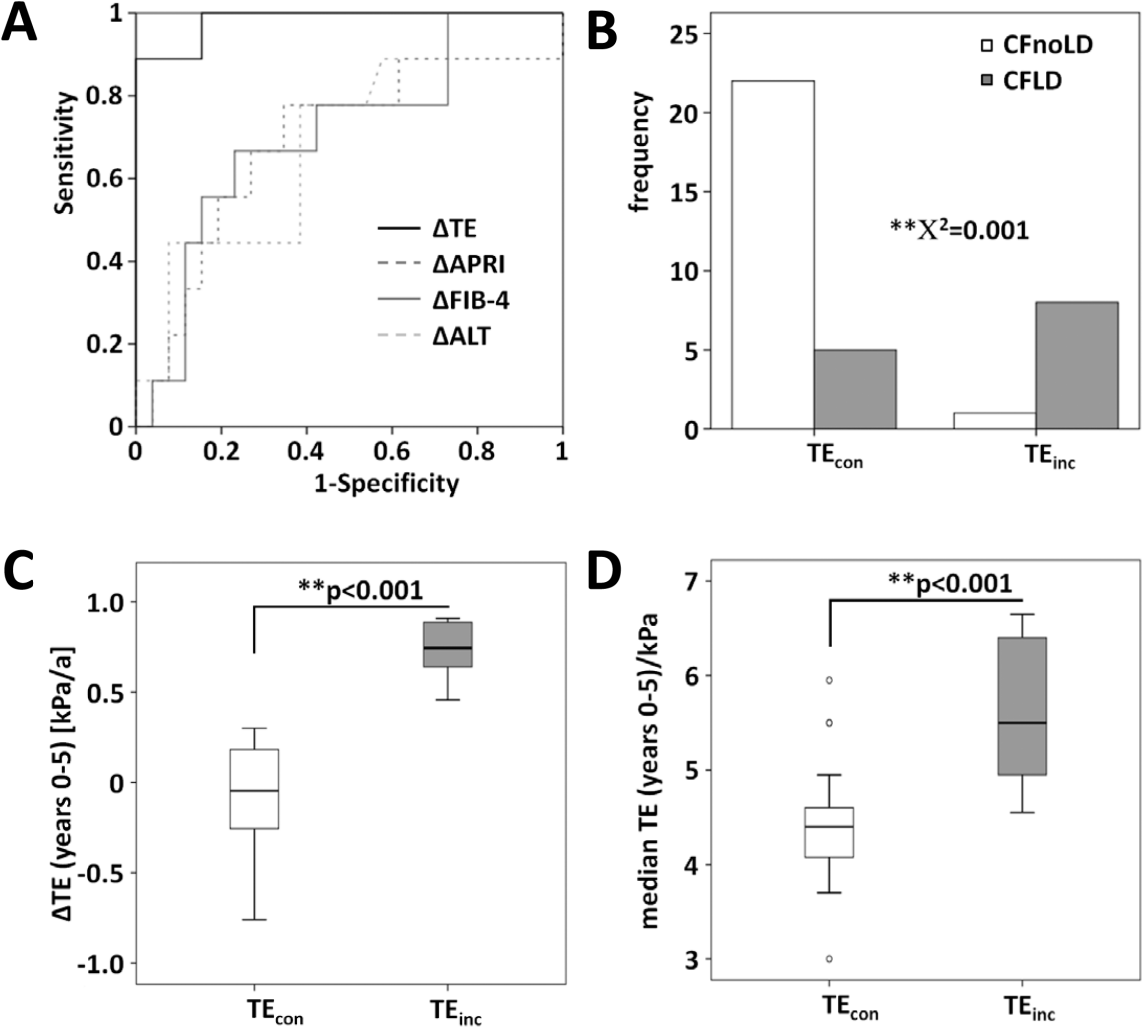
The increase in liver stiffness (ΔTE) defines the development of CFLD in pediatric patients. **(A)** The receiver operating characteristics (ROC) analysis identified a consistent increase of liver stiffness in 9 of 10 pediatric patients with a TE(4–5) >6.3 kPa and fixed the cut-off as rationale for grouping ΔTE_cut-off_ = 0.38 kPa/year. The frequency of CFLD **(B)**, the enhancement of liver stiffness ΔTE **(C)**, and the median stiffness **(D)** was increased in the TE_inc_-group compared to the TE_con_-group.

10 (28%) out of 36 pediatric CF patients had a final stiffness above the threshold of 6.3 kPa in the fourth and fifth year. In order to identify those patients with a continuous rise of liver stiffness and final stiffness above the threshold of 6.3 kPa, the optimal cut-off for the mean increase in TE was calculated by ROC analysis to be ΔTE_cut-off_ = 0.38kPa/year (Fig 2A). Eventually, 9 (25%) out of 36 pediatric CF patients showed a mean increase in TE-value of >0.38kPa per year (group labelled TE_inc_ with ΔTE_inc_>0.38kPa/year). 27 pediatric CF patients (75%) exhibited a stable stiffness value below the threshold of 0.38 kPa/year. These patients were labelled TE_con_ and served as a control group for those 9 (25%) pediatric CF patients with a continuously increasing liver stiffness (target group TE_inc_). Fibrosis scores APRI and FIB-4 as well as clinical parameters specific for liver disease were assessed.

As shown in Table 1, patients with TE_inc_ and TE_con_ did not differ in the baseline demographics and clinical data such as age, BMI, or the involvement of the pancreas and liver enzyme levels. Furthermore, there was no difference in the liver stiffness and fibrosis scores at the beginning of the study between the patients with stable liver stiffness over time and those with exacerbation.

It is important to mention that when TE_con_ and TE_inc_ patients were stratified according to the criteria for CFLD diagnosis by Debray et al [8] and analyzed by Chi-square test, it showed that longitudinal liver stiffness measurements with TE were able to distinguish CFnoLD and CFLD with a high significant value of χ^2^ = 0.001(**). As illustrated in Fig 2B, within TE_con_ patients only 5 (18.5%) patients developed CFLD during the follow-up period, as diagnosed by guideline criteria. In contrast, out of the 9 patients within the TE_inc_ group, 8 patients (= 88.9%) were diagnosed with CFLD according to the criteria by Debray during the follow-up. Fig 2C and 2D demonstrate the differences in ΔTE and median TE(year 0–5) between TE_con_ and TE_inc_. The median values of the yearly increase of liver stiffness were ΔTE_inc_ = 0.82 kPa (0.46–1.79) and ΔTE_con_ = -0.05 kPa (-0.76–0.3) with *p*<0.001. Similarly, the difference in median stiffness definitely supported the distinction of the two groups: median TE_con_(year 0–5) = 4.5 kPa and median TE_inc_(year 0–5) = 5.45 kPa, *p*<0.001 (Fig 2D).

**Fig 3:**
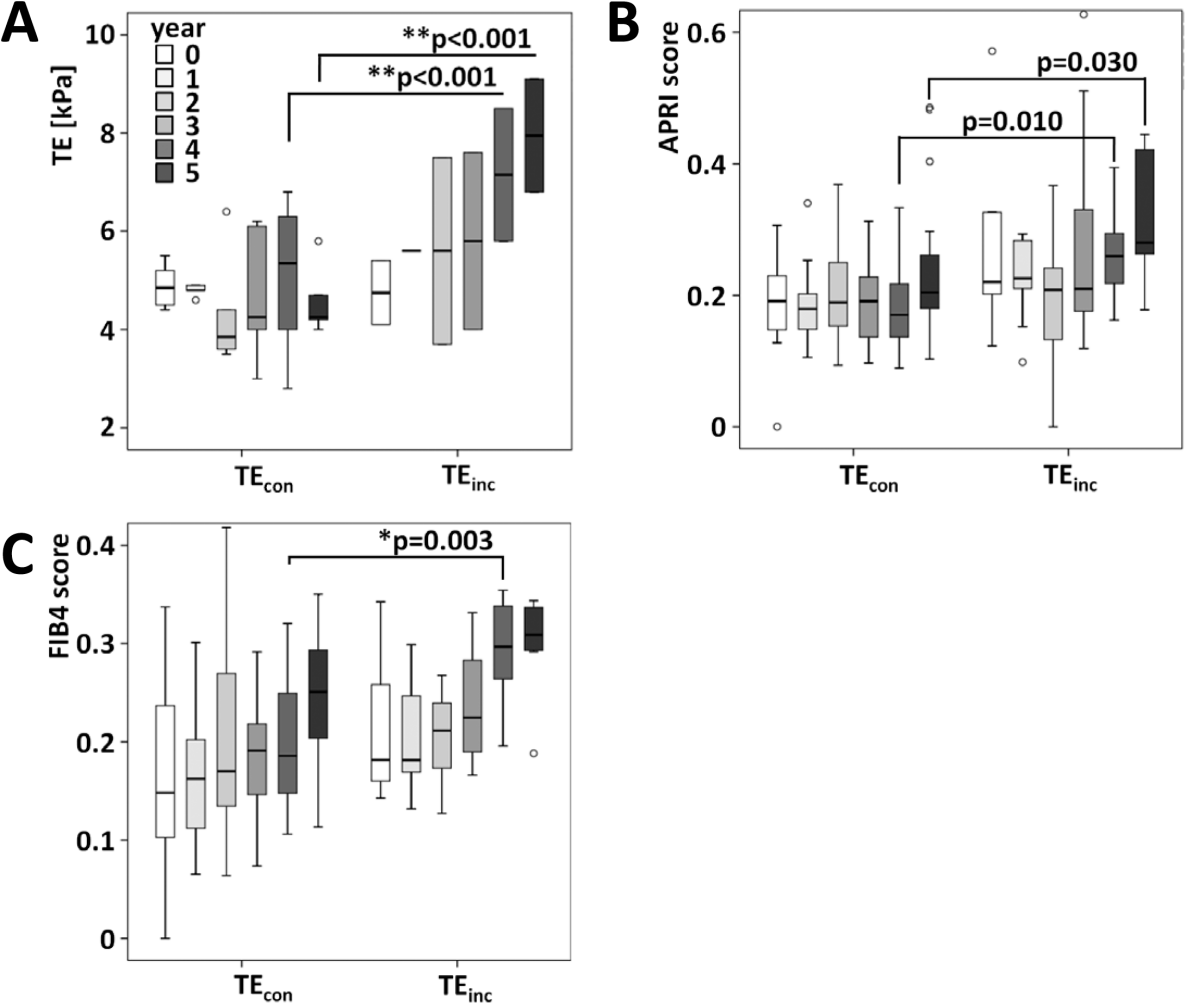
The longitudinal assessment of TE, APRI-score, and FIB-4-score indicated increased liver stiffness and fibrosis. **(A)** TE was enhanced in the TE_inc_-group 4 and 5 years after the first assessment. **(B)** Although the ANOVA calculated *p*-value was rather low in comparison of TE_inc_ and TE_con_ 4–5 years after the first examination, statistical significance was not reached. **(C)** FIB-4 was enhanced in patients of the TE_inc_-group 4 years after the first examination, however it did not reach statistical significance at the 5^th^ exploration. Interestingly, the FIB-4-score tends to increase gradually in patients of the TE_con_-group.

In the TE_inc_ group, the increase of liver stiffness was mostly pronounced and highly significant (*p*<0.001) in the fourth and fifth year of the follow-up (Fig 3A). This increase in liver stiffness was accompanied by an enhanced FIB-4 score with a median yearly increase of 0.03 (0–0.09, *p* = 0.003) at the fourth year of the follow-up while the APRI score increased tendentially but did not reach the level of significance (Fig 3B and 3C). Both, APRI and FIB-4, were positively correlated with liver stiffness (*r*_APRI-TE_ = 0.78, *p*<0.001 and *r*_FIB-4-TE_ = 0.75, *p*<0.001).

## Discussion

CFLD is a relatively frequent and an early complication of CF develops in 5–10% of children within their first decade of life. There is also a chance of 4.5% of an incidence of cirrhosis during a median period of 5 years from the time of the diagnosis of the liver disease [11].

Given these considerations, the early diagnosis of CFLD is urgently needed, and patients with an early stage liver disease are more likely to benefit from therapy with ursodeoxycholic acid (UDCA) [12]. Although defined criteria for the diagnosis and management of cystic fibrosis-associated liver disease have been published recently, they might miss early stages of CFLD, especially in the absence of significant fibrosis deposition within the liver [8]. As shown in previous reports from our group and others, the measurement of liver stiffness with transient elastography has a high diagnostic accuracy for the detection of CFLD in adults and children [4–6,12–14]. Thus, the approach of assessing of liver stiffness using TE sensitively and noninvasively, which directly reflects increased matrix deposition and fibrogenesis in the liver, can complement or even facilitate conventional clinically oriented diagnosis by enhancing specificity and improving early detection of CFLD.

Nevertheless, so far only two studies presented follow-up data for TE-assessment in CFLD patients [4,12]. Karlas et al. described a stable course of CFLD in adult CF patients once they have passed adolescence [4]. Van Biervliet et al. retrospectively showed stable liver stiffness in young CF patients without liver disease on the one hand, and progressively increasing stiffness in young CF patients with developing liver disease on the other hand [12]. Our current work is the first study to present prospective data showing that TE is a sensitive diagnostic tool for the identification of patients at risk for progressing CFLD. In this regard, we calculated a cut-off for the rise in liver stiffness of ≥0.38kPa/year to be optimal for the identification of children with progressively increasing liver stiffness.

8 out of 9 children of the TE_inc_-group were also identified to have CFLD ofy comprehensive conventional criteria [8]. Nevertheless, these criteria failed in one patient of the TE_inc_ group. According to previously published data [4], no increase in liver stiffness was observed in adults which underlines the hypothesis of a stable course of CFLD in the majority of adult CF patients once they have passed adolescence [15].

A recently published, liver biopsy based study showed that the biomarker scores APRI and FIB-4 differentiate between CFLD and CFnoLD in pediatric CFLD patients [2]. Therefore APRI appears superior to FIB-4 and furthermore exhibited a high accuracy in predicting severe liver fibrosis [2]. The positive correlation of APRI and FIB-4 with liver stiffness underlines that transient elastography performs diagnosing liver fibrosis in patients with CFLD.

A possible limitation of our study might be the lack of histopathologic assessment of CFLD in our cohort. However, due to the focal nature of CFLD, liver biopsy is controversially discussed in CFLD and thus not acting as regular diagnostic [8,16]. Moreover, it would have been desirable to include a higher number of patients in the pediatric cohort. Approximately 67% of patients with cystic fibrosis develop hepatic steatosis, but the pathogenic and histomorphologic relation of CFLD to NAFLD has not been investigated so far and the mechanism is not clearly understood [17,18]. It has been suggested that the high risk of CF patients to develop CF related diabetes is a prerequisite for hepatic steatosis [18]. Nevertheless, the different phenotypes of CFLD may not be uniformly detected by TE and could not be distinguished in the current study.

## Conclusion

This study can be distinguished from other studies, as it is a prospective long term observation of CF patients to be at risk for CFLD. Furthermore we compared TE with established histology proven fibrosis scores (APRI and FIB-4). Our newly described cut-off for pathologic increase of liver stiffness might enable to detect developing CFLD using consequent follow up TE measurements before reaching the level of stiffness indicating established CFLD. With respect to the limited size of the analyzed cohort, we perceive our study as a signal to encourage a prospective, multi-center, long term follow up study to confirm our data and to refine the suggested cut-off for the rise in liver stiffness for the identification of children at risk for development of CFLD.
